# A bioinspired configurable cochlea based on memristors

**DOI:** 10.3389/fnins.2022.982850

**Published:** 2022-10-03

**Authors:** Lingli Cheng, Lili Gao, Xumeng Zhang, Zuheng Wu, Jiaxue Zhu, Zhaoan Yu, Yue Yang, Yanting Ding, Chao Li, Fangduo Zhu, Guangjian Wu, Keji Zhou, Ming Wang, Tuo Shi, Qi Liu

**Affiliations:** ^1^Key Laboratory of Microelectronic Devices and Integrated Technology, Institute of Microelectronics, Chinese Academy of Sciences, Beijing, China; ^2^Frontier Institute of Chip and System, Fudan University, Shanghai, China; ^3^School of Integrated Circuits, University of Chinese Academy of Sciences, Beijing, China; ^4^Zhejiang Laboratory, Hangzhou, China; ^5^State Key Laboratory of Integrated Chips and Systems, Fudan University, Shanghai, China; ^6^School of Integrated Circuit, Anhui University, Hefei, China

**Keywords:** cochlea, configurable, memristor, filter, speech recognition

## Abstract

Cochleas are the basis for biology to process and recognize speech information, emulating which with electronic devices helps us construct high-efficient intelligent voice systems. Memristor provides novel physics for performing neuromorphic engineering beyond complementary metal-oxide-semiconductor technology. This work presents an artificial cochlea based on the shallen-key filter model configured with memristors, in which one filter emulates one channel. We first fabricate a memristor with the TiN/HfO_x_/TaO_x_/TiN structure to implement such a cochlea and demonstrate the non-volatile multilevel states through electrical operations. Then, we build the shallen-key filter circuit and experimentally demonstrate the frequency-selection function of cochlea’s five channels, whose central frequency is determined by the memristor’s resistance. To further demonstrate the feasibility of the cochlea for system applications, we use it to extract the speech signal features and then combine it with a convolutional neural network to recognize the *Free Spoken Digit Dataset*. The recognition accuracy reaches 92% with 64 channels, compatible with the traditional 64 Fourier transform transformation points of mel-frequency cepstral coefficients method with 95% recognition accuracy. This work provides a novel strategy for building cochleas, which has a great potential to conduct configurable, high-parallel, and high-efficient auditory systems for neuromorphic robots.

## Introduction

**FIGURE 1:**
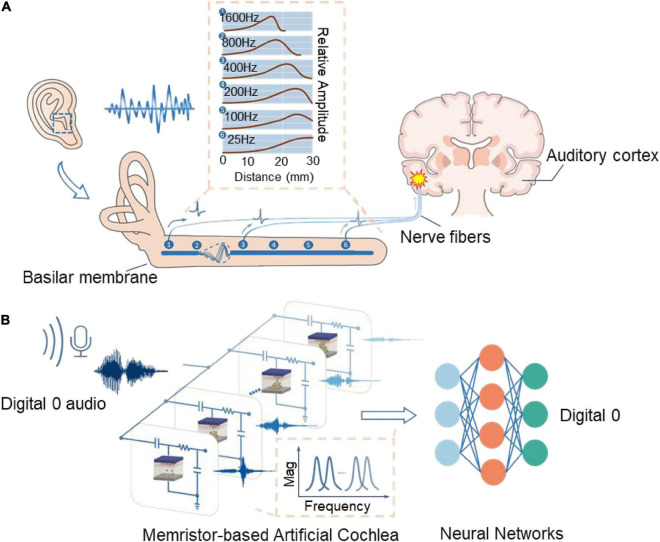
Biological vs. bioinspired cochlear auditory recognition system. **(A)** Schematic of speech recognition in the biological cochlea system. **(B**) Speech signal recognition system using the memristor-based artificial cochleas.

Speech, as one of the most important sensory information, plays a critical role in human activities, such as communication, interaction, danger warnings et al. The cochlea is the core element of receiving and preprocessing the voice signal, which generates sparse voice spikes and transmits them to the auditory cortex for further recognition. In cochlea, the vibration of sound causes the hair cells to bend, which in turn causes the graded receptor potential on the hair cells (Nelken, 2020; Caprara and Peng, 2022). The hair cells on the basilar membrane with different locations have their specific response behavior, endowing them with band-pass filtering capability and generating specific electrical signals. The electrical signals will be transmitted to the lower ventral cochlear nucleus in parallel through multiple auditory fiber channels, finishing the first step of speech signal feature extraction (Pyott and von Gersdorff, 2020; Marin et al., 2022), as shown in Figure 1A. Benefit from the multi-channel parallel processing feature, the organisms could process and perceive complex audio signals with high efficiency (Luo, 2021). Inspired by the biological cochlea, artificial cochleas have been widely used in mobile devices (Geronazzo et al., 2020; Zheng et al., 2021), smart homes (Mondal and Barman, 2022; Priya et al., 2022), biomedical healthcare system (Islam et al., 2022; Wang et al., 2022), and other voice interaction interfaces to perform assigned tasks (Eichenauer et al., 2021; Ghosh et al., 2022). However, the artificial cochlear system based on complementary metal-oxide-semiconductor (CMOS) technology, with the advent of the post-Moore era, presents challenges on system complexity, energy consumption, scalability and configurability (Xu et al., 2018; Wang et al., 2021; Ding et al., 2022). Therefore, developing novel devices or circuits with new principle to build artificial cochlea deserves more attention and is becoming a hot topic in this field.

Shintaku et al. (2010) developed an artificial basilar membrane with a flexible PVDF thin film acoustic sensor that was configured with multiple electrodes. The constructed sensor features a frequency-selection response due to the piezoelectric effect, and the frequency response of 3.64, 2.32, and 1.88 kHz channels are experimentally demonstrated. Also, Jang et al. (2015) developed a piezoelectric artificial basement membrane (ABM) to imitate signal handling in cochlea and achieved sound response varying from 2.92 to 12.6 kHz. However, the dimension of novel devices is on a scale of mm or cm. These sensors’ sound response frequency range is narrow and the number of achieved channels is relatively limited, making them difficult to accomplish the preprocessing process of complex sound signals. The filter bank is one of the most common strategies to emulate the basement membrane’s (BM) characteristics in the biological auditory systems, in which each band-pass filter has its specific central frequency. In hardware implementations, the potentiometer is generally used to serve as a variable resistor in band-pass filter, which faces the problem of limited programmability (Hill et al., 1968; Gao et al., 2020) or complicated circuits that consumes much area and power (Xu et al., 2018; Farhadi et al., 2020; Wang et al., 2021). Memristor [or resistive random access memory (RRAM)], as an emerging non-volatile memory, possess high reconfigurability, low power consumption, and high-density characteristics. These features make it provide a novel physical basis for constructing artificial auditory systems (Gao et al., 2022; Zhong et al., 2022). Although memristor-based Sallen-Key circuit with tunable gain-bandwidth and center frequency characteristics have been proposed for emulating the cochlea, it still lack experimental demonstration based on memristors’ multi-levels (Li et al., 2020; Barraj et al., 2021; Onyejegbu et al., 2022). In addition, Wu et al. (2021) used the stochastic gradient descent-supervised learning rule to train the preprocessed audio features. The weights were mapped into W/MgO/SiO_2_/Mo memristor arrays to complete the speech classification tasks. Gao et al. (2022) transformed the binaural soundwaves to Fourier domain at first and then experimentally verified *in situ* learning of the sound localization function in 1K HfO_x_ memristor array. However, they emphasize the emulation of the auditory cortex’s function, ignoring the implementation of the cochlea’s filtering function.

In this work, we propose an artificial cochlea based on shallen-key filter model configured with memristors. The memristor has the structure of TiN/HfO_x_/TaO_x_/TiN and features a multilevel analog resistive state, making it suitable for serving as the configurable potentiometer. Combining the memristor, we build a shallen-key filter circuit to implement the cochlea function, as shown in Figure 1B. By programming the memristor into different resistance value, the artificial cochlea could output signals with specific frequencies and gains. Using such a cochlea circuit, we experimentally demonstrated the filtering behavior of 5 channels with different central frequencies. Finally, we connect the circuits with a convolutional neural network (CNN) to recognize 10 class digital radio in the *Free Spoken Digit Dataset*, achieving 92% accuracy under the case of 64 cochlea’s channels. The results show that the proposed cochlea system could compete with the mel-frequency cepstral coefficients (MFCC) method of extracting the speech features, illustrating the feasibility of constructing high-efficient artificial cochlea systems based on memristors.

## Materials and methods

### Device fabrication

The detailed fabrication processes of the memristors are as follows. First, the 30 nm TiN bottom electrode is deposited with physical vapor deposition. After that, HfO_x_ and TaO_x_ is stacked up by atomic layer deposition method, in which the thickness of HfO_x_ is 8 nm, and TaO_x_ is 45 nm. Then, the top electrode TiN is grown by the physical vapor deposition to 30 nm. The transistor in the 1T1R structure is used to obtain expected memristor conductance states through limiting the current by adjusting gate voltage (Lu et al., 2020). The transistor is built on a standard 0.18 μm CMOS foundry process technology node by the Semiconductor Manufacturing International Corporation (SMIC).

### Measurement methods

After FIB etching technique (FEI Helios Nanolab 450s, UK) for thinning the samples, the TEM images and EDS line scan/mapping composition analyse were operated by JEOL ARM 200F cold field emission gun TEM/STEM with cs-corrector under 200 kV voltage. The electrical characteristics of the 1T1R were obtained from Agilent B1500A Semiconductor Device Analyzer using DC sweep module or waveform generator/fast measurement unit module (WGFUM) at room temperature. The memristor-based cochlea circuit was constructed on a printed circuit board (PCB). During the circuit test, a Keysight 81160A pulse generator was served as the power source, and a Keysight Infinii Vision MSO-X 3104T oscilloscope was chosen to monitor output signals. The neural network simulation in speech recognition task was implemented in the Python platform.

## Results

### Memristive device

**FIGURE 2:**
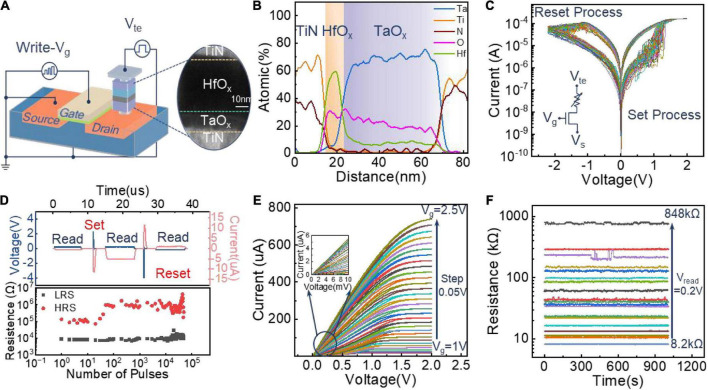
Device structure and electrical properties. **(A)** Film-stacked structure of 1T1R, consisting of a transistor and TiN/HfO_x_ /TaO_x_ /TiN with a TEM image. **(B)** Low panel quantifies the atomic profile of primary elements across the memristor from the EDS line scan upper panel. **(C)** The I-V characteristics of the 1T1R in 100 repeated DC sweeps during the set/reset processes. For the set process, the scan voltage of 0–1.7 V is applied to the TE with the voltage of 1.5 V applied to the gate; for the reset process, the scan voltage of 0–2.2 V is applied to the SE with the voltage of 4 V applied to the gate. **(D)** The I-V electrical characteristics of the device under pulsed scanning with set/reset process. During the set process, a pulse (2.2 V, 100 ns) is applied to TE terminal with V_g_ = 4 V; during the reset process, a pulse (4 V,100 ns) is applied to SE terminal with V_g_ = 4 V. Endurance results show the reliable HRS and LRS up to 5 × 10^5^ cycles. **(E)** Multilevel resistance programming characteristic of the device under DC sweep. V_g_ increases from 1 to 2.5 V with a 0.05 V step. The inset shows the good linearity of the memristor under 0–0.1 V sweeping on TE. **(F)** Multi-resistance stability retention characteristics of the device.

The structure of the fabricated memristor is shown in Figure 2A, configured with TiN/HfO_x_ /TaO_x_ /TiN. The inset depicts the stacked thin films by the high-resolution TEM (HRTEM) images. When different voltage stimuli is applied to the memristor during the set/reset process, the HfO_x_ layer serves as the functional layer because of the changing morphology of the conductive filament (Zhang et al., 2021). The TaO_x_ layer works as a built-in compliance layer, stabilizing the injected current in both forming and programming operation, leading to a uniform LRS distribution (Lin et al., 2021). The flexible and configurable characteristics of memristors are the basis of building an artificial cochlea system. To better understand the composition of the memristor, the lateral composition distribution of the designed TiN/HfO_x_/TaO_x_/TiN is analyzed, as shown in Figure 2B. The atomic percentages of the main element in each position confirm the concentration and distribution of ingredients, consolidating the reprocess of the same migration species in conductive filaments (Chang et al., 2018). We then perform the typical DC sweep to verify the analog switching behavior. Initially, the device is in a high-resistance state (HRS). Before presenting a normal switching behavior, a forming operation is conducted with a gate voltage of 1.2 V and a scanning voltage from 0 to 4 V (see Supplementary Figure 1). Figure 2C shows 100 continuous switching cycles, observing that the device has good resistance state uniformity.

To further demonstrate the switching speed of the memristor, we conducted the pulse measurement on the device, as shown in Figure 2D. Before performing the testing, the device is set to an HRS. Then, a SET pulse (t_w_ = 100 ns, V_te_ = 2.2 V) is applied to the TE terminal to conduct the SET operation with V_g_ = 1.5 V on the gate terminal of the 1T1R. During carrying out the reset operation, a RESET pulse (t_w_ = 7.5 us, V_s_ = 4 V) is applied on the SE terminal with V_g_ = 4 V. To monitor the resistance state, a read pulse (t_w_ = 7.5 us, V_te_ = 0.2 V) is applied on the TE terminal with V_g_ = 1.5 V. It can be seen that the device is successfully switched between HRS and low resistance state (LRS) with a switching time of less than 100ns. And the device works well after 5 × 10^4^ cycles. Then, to prove the programmable capability of the memristor, we test the multilevel resistance characteristics under different V_g_ voltages during the set process, as shown in Figure 2E. With increasing of the V_g_, the compliance current increases, which induces a lower resistance value of the memristor. The sweeping voltage in reset process increases when the memristor is programmed into a lower LRS, as shown in Supplementary Figure 2. Besides, the multilevel resistance characteristics can also be obtained by increasing the sweeping voltage on SE during the reset process, as shown in Supplementary Figure 3. The results show that the fabricated memristor features excellent multilevel resistance characteristics. Finally, to investigate the stability of memristor’s multilevel behaviors, we test the retention performance of multilevel resistance obtained under different compliance currents, as shown in Figure 2F. The results show that the device maintains stable resistance states over 10^3^s, proving the feasibility of the memristor as a configurable potentiometer in the filter circuit.

### Filter circuit based on memristor

**FIGURE 3:**
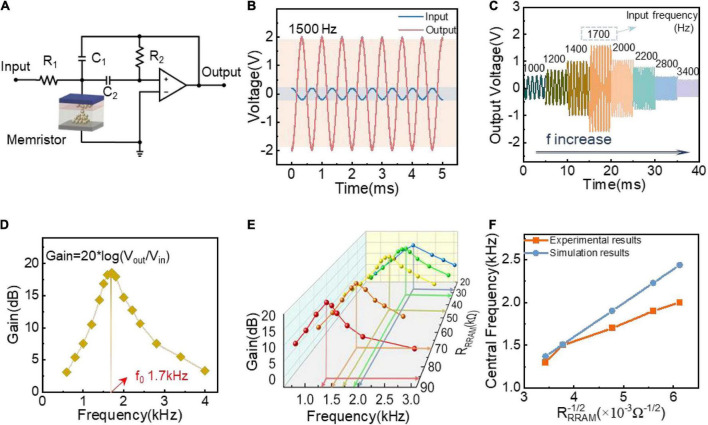
Bioinspired cochlea filter circuit and experimental results. **(A)** Circuit structure of bioinspired cochlea filter circuit based on memristor, where R1 = 1 MΩ, R2 = 100 MΩ, C1 = C2 = 40 pF. **(B)** The output response characteristics when the sinusoidal signal (0.2 V, 1,500 Hz) input to the circuit with the 44 kΩ memristor’s resistance. **(C)** Output signals when the input sinusoidal signal’s frequency increases from 1,000 to 3,400 Hz. **(D)** The amplitude-frequency characteristic curve of the memristor-based circuit when the memristor is programmed to 44 kΩ. **(E)** Multiple amplitude-frequency characteristic curves of the memristor-based filter circuit when the memristor is programmed to 86, 70, 44, 32, 26.7 kΩ, respectively. **(F)** Comparison diagram of the relationship between f_0_ and ${\text{R}}_{\text{RRAM}}^{-1/2}$ extracted from experimental and simulation results.

To further emulate the filter function of the cochlea based on the constructed memristors, we introduce a shallen-key circuit that consists of an op-amp, two capacitors, two resistors, and a memristor, as shown in Figure 3A. First, we developed a circuit model to illustrate the effect of the memristor’s resistance state on the circuit’s amplitude-frequency response. According to the Kirchhoff’s law, the transfer function can be obtained as follows (Kugelstadt, 2009):


(1) $$A\,\left(j\,\varphi \right)=A_{m}\times \frac{\frac{1}{Q}\,j\,\varphi }{1+\frac{1}{Q}\,j\,\varphi +{\left(j\,\varphi \right)}^{2}}$$

Where φ = w/w_0_,*w* is the angular frequency of the input signal and w_0_ = 2πf_0_ is the center angular frequency. Besides, the transfer function $\dot{\text{A}}\,\left(\mathrm{jw}\right)$ is related to A_m_, Q and the frequency of the input signal, which represents the response feature between output signals and input signals of the filter circuit.

*A_m* represents the amplitude ratio of output and input signals, which is formulated as:


(2) $$A_{m}=-\frac{R_{2}}{R_{1}}\times \frac{C_{2}}{C_{1}+C_{2}}$$

The latter part of the Formula 1 in the transfer function represents the phase relationship between the output signal and the input signal. In which Q is the quality factor that characterizes the ability to distinguish adjacent frequency components in the signal. The higher Q means the stronger filter ability to distinguish signal frequency. The expression of Q is as follows:


(3) $$\frac{1}{Q}=\left(C_{1}+C_{2}\right)\,\sqrt{\frac{R_{1}//R_{\mathrm{Memristor}}}{R_{2}\,C_{1}\,C_{2}}}$$

The circuit has maximum output amplitude when the input signal’s frequency is f_0_, which called center frequency and f_0_is derived as follows:


(4) $$f_{0}=\frac{1}{2\,\pi \,C}\,\sqrt{\frac{1}{R_{2}}\,\left(\frac{1}{R_{1}}+\frac{1}{R_{\mathrm{Memristor}}}\right)}$$

where *C*_1_ = *C*_2_ = *C*.

According to the Formula 4, we can obtain that as the resistance state of the memristor decreases, the center frequency f_0_increases, which enables the memristor-based filter with different center frequency f_0_ when the memristor’s resistance state changes. This behavior is just like the filtering characteristics of the basilar membrane at different positions (Areias et al., 2021; Yao et al., 2022). The trendency can be explained by the fact that the current flowing through the R_1_ is divided into the current flowing through C_1_, C_2_ and the memristor. When the resistance state of the memristor decreases, the current flowing through both C_1_ and C_2_ decreases, which results in lower output amplitude. Since the equivalent impedance of the capacitor is inversely proportional to signal frequency, the center frequency f_0_increases when R_Memristor_ is adjusted to a lower value. Hence, there is a specific center frequency f_0_ corresponding to different memristor resistance state. This is essential working principle for the realization of the memristor-based configurable artificial cochlea.

To confirm the filtering properties of memristor-based cochlea circuit, the output response is tested with memristor programmed to 44 kΩ. When a sinusoidal signal (0.2 V, 1,500 Hz) is applied to the circuit, the output signal’s amplitude is 2 V, as shown in Figure 3B. The result shows that the cochlea circuit has amplification function when input signal’s frequency is 1,500 Hz. To elaborately investigate the amplitude-frequency characteristics of the circuit, the sinusoidal signal with identical amplitude but different frequencies is applied to the circuit in turn, and the results are shown in Figure 3C. Obviously, with increasing of the frequency, the output voltage amplitude increases at first, then decreases. There is a maximum value when the input frequency is 1,700 Hz, which is the so-called central frequency. To more intuitively obtain the response curve of the cochlea under different input frequency, the gain value (ratio of output amplitude to input amplitude) extracted from Figure 3C is shown in Figure 3D. We clearly observe that the gain value increase firstly then decreases with the increasing of input’s frequency, demonstrating that the cochlea circuit possess good frequency-selection characteristic. Besides, we illustrate that the cochlea has different amplitude-frequency characteristics when memristor programmed to different resistance states, as depicted in Figure 3E. As memristor’s resistance value decreases, the circuit’s center frequency f_0_ increases. Therefore, we can configure the frequency-selection characteristic (f_0_) of the cochlea circuit by programming the memristor with different resistance values.

We further replot the relationship curve between f_0_ and${\text{R}}_{\text{RRAM}}^{-1/2}$, which is extracted from Figure 3E, as shown in Figure 3F. The center frequency f_0_ follows the sub-linear function of ${\text{R}}_{\text{RRAM}}^{-1/2}$, which is consistent with the relationship derived from Formula 4. What’s more, the experimental f_0_-${\text{R}}_{\text{RRAM}}^{-1/2}$ curve is slightly lower than the ideal simulation results. This is because of the non-linear I-V characteristics of the memristor device. The higher the resistance, the higher the non-linearity, which results in higher deviations. What’s more, the wiring connection may introduce parasitic capacitance during the experimental test. These two reasons make the center frequency in the experimental result smaller than the simulated result.

### Speech recognition with bionic cochlear system

**FIGURE 4:**
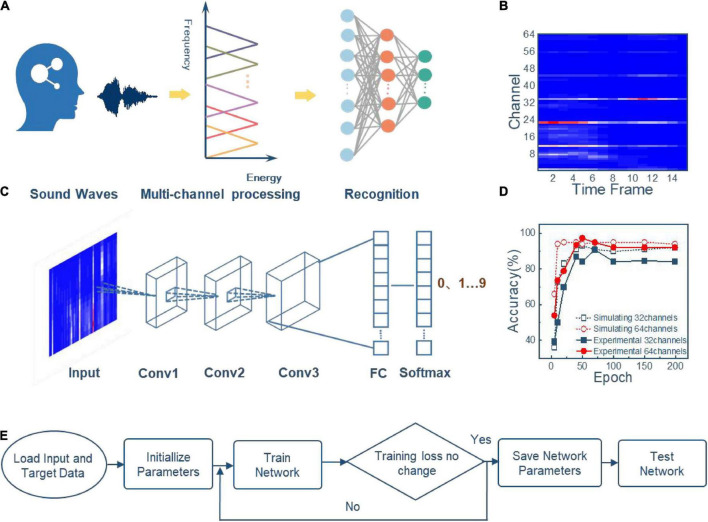
Zero to nine digital audio recognition realized in the artificial cochlear system based on CNN neural network. **(A)** Illustration of the artificial cochlea speech recognition system. **(B)** Energy spectrum of digital 0 speech signal after feature extraction by 64-channel parallel filter circuits. **(C)** Schematic diagram of CNN speech signal recognition. **(D)** Network simulation flow chart. **(E)** Experimental and simulated recognition accuracy of 10 digital speech audio recognition under 32 and 64 channels.

In biology, the electrical signals generated at the basement membrane will be projected to the cortex layer for advanced cognitive analysis (Elgoyhen, 2020; Nelken, 2020). In the neuromorphic system, neural networks are usually used to emulate the cortex for performing intelligent tasks (Zhang et al., 2020; Zhu et al., 2022). To verify the speech processing ability of the artificial cochlea system, a CNN is introduced to complete the following recognition tasks. In the artificial cochlea auditory recognition system, the audio voltage signal is input into the cochlea multiple memristor-based filter circuits for preprocessing, and the feature extraction result is input to CNN for recognition, as shown in Figure 4A. By modulating the memristors’ states in 64 filter channels into different resistance values, we obtain 64 central frequencies that corresponds to 64 Fourier transform transformation points in the conventional methods. Because of the reconfigurability of the memristor, the constructed filters consumes less hardware overhead than the conventional methods that use the complex potentiometers (Adesina et al., 2021; Wang et al., 2021).

Take the digital 0 audio signal as an example, the signal processing flow is illustrated as follows: (1) The audio signal voltage is input into the 64-channel bioinspired cochlea memristor filter circuits in parallel, then the filtered signals with different frequency features are obtained; (2) Divide the output signals into overlapping 15 frames and compress signals in each frame. The obtained energy spectrum is shown in Figure 4B, which will be further processed by a Mel non-linear processing unit. (3) The energy spectrum is input to the CNN network for classification.

The used CNN consists of an input layer, three convolutional layers, one fully connected layer, and one output layer, as shown in Figure 4C. The 500 audios from the Free Spoken Digit Dataset are used to verify digital speech recognition’s ability of the bioinspired cochlea system. Four hundred and fifty audios are used for network training to extract model parameters, and the remaining 50 audios are used for testing. Figure 4D presents the training and testing processes of the CNN. The simulated and experimental results with 32 and 64 channels are shown in Figure 4E. After 200 iterations, the recognition accuracy of the 64-channel artificial cochlea system is 92%, which is compatible with 95% accuracy that utilizes the MFCC scheme with traditional 64 fourier transform transformation points. The former method for processing speech signals with analog filter circuit is proved to be more energy efficient (Giraldo et al., 2020; Wang et al., 2021).

We also found that accuracy of the 64-channel artificial cochlea system is higher than that in an artificial cochlea system with 32-channels (84%). It can be explained that a larger number of channels extract more frequency features, which is beneficial to enhance the network performance. The results demonstrate that the proposed artificial cochlea in this work offers a potential strategy to construct intelligent audio systems and conduct speech tasks.

## Discussion

In summary, we built an artificial cochlea based on TiN/HfO_x_/TaO_x_/TiN memristors and shallen-key filter model to implement the processing procedure of speech information in the mammalian cochlea. Because of the programmable non-volatile multilevel resistances of the memristor, the constructed artificial cochlea is configurable and flexible. Depending on the resistance state of the memristor, each channel of the cochlea possessed its own central frequency, which was successfully demonstrated in the experiment. To present the practical applications of the artificial cochlea system, we further combine it with a CNN to identify 10 classes of audio signals in the *Free Spoken Digit Dataset*. The results show that the recognition accuracy reaches 92% when the cochlea has 64 memristor-based filtering channels. This work presents a promising way of building configurable artificial cochlea with memristors and has a great potential for robotic sensing applications.

## Data availability statement

The original contributions presented in this study are included in the article/Supplementary material, further inquiries can be directed to the corresponding author.

## Author contributions

LC and XZ designed the experiments, conducted the electrical measurement, and prepared the manuscript. LC and LG conducted the simulation. XZ fabricated the 1T1R device. ZW contributed to EDS and TEM. XZ and QL supervised the research. All authors discussed the data, revised the text, and approved the submitted version.

## References

[B1] Adesina N. O. Srivastava A. Khan M. A. U. (2021). “Evaluating the performances of memristor, FinFET, and graphene TFET in VLSI circuit design,” in *Proceedings of the 2021 IEEE 11th annual computing and communication workshop and conference*, Nevada, CA, 0591–0595. 10.1109/CCWC51732.2021.9376125

[B2] Areias B. Parente M. Gentil F. Jorge R. N. (2021). Influence of the basilar membrane shape and mechanical properties in the cochlear response: A numerical study. *Proc. Instit. Mech. Eng. Part H J. Eng. Med.* 235 743–750. 10.1177/09544119211003443 33749399

[B3] Barraj I. Bahloul M. A. Masmoudi M. (2021). Design of 3–5 GHz tunable memristor-based OOK-UWB transmitter. *AEU Int. J. Electron. Commun.* 132:153664. 10.1016/j.aeue.2021.153664

[B4] Caprara G. A. Peng A. W. (2022). Mechanotransduction in mammalian sensory hair cells. *Mol. Cell. Neurosci.* 120:103706. 10.1016/j.mcn.2022.103706 35218890 PMC9177625

[B5] Chang C.-F. Chen J.-Y. Huang G.-M. Lin T.-Y. Tai K.-L. Huang C.-Y. (2018). Revealing conducting filament evolution in low power and high reliability Fe3O4/Ta2O5 bilayer RRAM. *Nano Energy* 53 871–879. 10.1016/j.nanoen.2018.09.029

[B6] Ding Y. Zhang Y. Zhang X. Chen P. Zhang Z. Yang Y. (2022). Engineering spiking neurons using threshold switching devices for high-efficient neuromorphic computing. *Front. Neurosci.* 15:1662–4548. 10.3389/fnins.2021.786694 35069102 PMC8766734

[B7] Eichenauer A. Baumann U. Stöver T. Weissgerber T. (2021). Interleaved acoustic environments: Impact of an auditory scene classification procedure on speech perception in cochlear implant users. *Trends Hear.* 25:23312165211014118. 10.1177/23312165211014118 34028332 PMC8150447

[B8] Elgoyhen A. B. (2020). Cochlear efferent innervation: Function, development and plasticity. *Curr. Opin. Physiol.* 18 42–48. 10.1016/j.cophys.2020.07.020

[B9] Farhadi M. Abbaspour-Gilandeh Y. Mahmoudi A. Mari Maja J. (2020). An integrated system of artificial intelligence and signal processing techniques for the sorting and grading of nuts. *Appl. Sci.* 10:3315. 10.3390/app10093315

[B10] Gao B. Zhou Y. Zhang Q. Zhang S. Yao P. Xi Y. (2022). Memristor-based analogue computing for brain-inspired sound localization with in situ training. *Nat. Commun.* 13:2026. 10.1038/s41467-022-29712-8 35440127 PMC9018844

[B11] Gao C. Wang H. Zhu Z. Zhang L. Yang Y. Cao G. (2020). A high-performance memristor device and its filter circuit application. *Phys. Status Solidi Rapid Res. Lett.* 14:2000389. 10.1002/pssr.202000389

[B12] Geronazzo M. Vieira L. S. Nilsson N. C. Udesen J. Serafin S. (2020). Superhuman hearing – Virtual prototyping of artificial hearing: A case study on interactions and acoustic beamforming. *IEEE Trans. Visual. Comput. Graph.* 26 1912–1922. 10.1109/TVCG.2020.2973059 32070968

[B13] Ghosh R. Ali H. Hansen J. H. L. (2022). CCi-MOBILE: A portable real time speech processing platform for cochlear implant and hearing research. *IEEE Trans. Biomed. Eng.* 69 1251–1263. 10.1109/TBME.2021.3123241 34705633 PMC8918373

[B14] Giraldo J. S. P. Lauwereins S. Badami K. Verhelst M. (2020). Vocell: A 65-nm speech-triggered wake-up SoC for 10- $\mu$ W keyword spotting and speaker verification. *IEEE J. Solid State Circuits* 55 868–878. 10.1109/JSSC.2020.2968800

[B15] Hill F. J. McRae L. P. McClellan R. P. (1968). Speech recognition as a function of channel capacity in a discrete set of channels. *J. Acoust. Soc. Am.* 44 13–18. 10.1121/1.1911047 5659828

[B16] Islam R. Abdel-Raheem E. Tarique M. (2022). A novel pathological voice identification technique through simulated cochlear implant processing systems. *Appl. Sci.* 12:2398. 10.3390/app12052398

[B17] Jang J. Lee J. Woo S. Sly D. J. Campbell L. J. Cho J. H. (2015). A microelectromechanical system artificial basilar membrane based on a piezoelectric cantilever array and its characterization using an animal model. *Sci. Rep.* 5:12447. 10.1038/srep12447 26227924 PMC4521187

[B18] Kugelstadt T. (2009). “Chapter 20 – Active filter design techniques,” in *Op amps for everyone*, 3rd Edn, eds Mancini R. Carter B. (Boston, MA: Newnes), 365–438. 10.1201/9781315152592-14

[B19] Li D. Zhang J. Yu D. Xu R. Iu H. H. C. Fernando T. (2020). A family of binary memristor-based low-pass filters with controllable cut-off frequency. *IEEE Access.* 8 60199–60209. 10.1109/access.2020.2982977

[B20] Lin H. Wu Z. Liu L. Wang D. Zhao X. Cheng L. (2021). Implementation of highly reliable and energy efficient in-memory hamming distance computations in 1 Kb 1-transistor-1-memristor arrays. *Adv. Mater. Technol.* 6:2100745. 10.1002/admt.202100745

[B21] Lu J. Wu Z. Zhang X. Wei J. Fang Y. Shi T. (2020). Quantitatively evaluating the effect of read noise in memristive hopfield network on solving traveling salesman problem. *IEEE Electron Device Lett.* 41 1688–1691. 10.1109/LED.2020.3021593

[B22] Luo L. (2021). Architectures of neuronal circuits. *Science* 373:eabg7285. 10.1126/science.abg7285 34516844 PMC8916593

[B23] Marin N. Lobo Cerna F. Barral J. (2022). Signatures of cochlear processing in neuronal coding of auditory information. *Mol. Cell. Neurosci.* 120:103732. 10.1016/j.mcn.2022.103732 35489636

[B24] Mondal S. Barman A. D. (2022). Human auditory model based real-time smart home acoustic event monitoring. *Multimedia Tools Appl.* 81 887–906. 10.1007/s11042-021-11455-1

[B25] Nelken I. (2020). From neurons to behavior: The view from auditory cortex. *Curr. Opin. Physiol.* 18 37–41. 10.1016/j.cophys.2020.07.019

[B26] Onyejegbu E. Zhumabay Z. Marzuki A. Ukaegbu I. A. (2022). A variable bandwidth memristor-based legendre optimum low pass filter for radio frequency applications. *Eng. Rep.* e12513. 10.1002/eng2.12513

[B27] Priya S. S. Aramoti S. Fathima S. (2022). “Home automation by speech detection system using deep learning,” in *Proceedings of the 2022 international conference on smart technologies and systems for next generation computing*, Villupuram. 10.1109/ICSTSN53084.2022.9761303

[B28] Pyott S. J. von Gersdorff H. (2020). “Book auditory afferents: Sound encoding in the cochlea,” in *The senses: A comprehensive reference*, ed. Fritzsch B. (Amsterdam: Elsevier), 487–500. 10.1016/B978-0-12-805408-6.00030-0

[B29] Shintaku H. Nakagawa T. Kitagawa D. Tanujaya H. Kawano S. Ito J. (2010). Development of piezoelectric acoustic sensor with frequency selectivity for artificial cochlea. *Sens. Actuat. A Phys.* 158 183–192. 10.1016/j.sna.2009.12.021

[B30] Wang D. Kim S. J. Yang M. Lazar A. A. Seok M. (2021). “9.9 a background-noise and process-variation-tolerant 109nW acoustic feature extractor based on spike-domain divisive-energy normalization for an always-on keyword spotting device,” in *Proceedings of the 2021 IEEE international solid- state circuits conference*, San Francisco, CA, 160–162. 10.1109/isscc42613.2021.9365969

[B31] Wang W. Pang J. Su J. Li F. Li Q. Wang X. (2022). Applications of nanogenerators for biomedical engineering and healthcare systems. *InfoMat* 4:e12262. 10.1002/inf2.12262

[B32] Wu X. Dang B. Wang H. Wu X. Yang Y. (2021). Spike-enabled audio learning in multilevel synaptic memristor array-based spiking neural network. *Adv. Intell. Syst.* 4:2100151. 10.1002/aisy.202100151

[B33] Xu Y. Thakur C. S. Singh R. K. Hamilton T. J. Wang R. M. van Schaik A. (2018). A FPGA implementation of the CAR-FAC cochlear model. *Front. Neurosci.* 12:198. 10.3389/fnins.2018.00198 29692700 PMC5902704

[B34] Yao W. Liang J. Ren L. Ma J. Zhao Z. Wang J. (2022). Revealing the actions of the human cochlear basilar membrane at low frequency. *Commun. Nonlinear Sci. Numer. Simul.* 104:106043. 10.1016/j.cnsns.2021.106043

[B35] Zhang X. Wu Z. Lu J. Wei J. Lu J. Zhu J. (2020). “Fully memristive SNNs with temporal coding for fast and low-power edge computing,” in *Proceedings of the 2020 IEEE international electron devices meeting*, San Francisco, CA. 10.1109/IEDM13553.2020.9371937

[B36] Zhang Y. Mao G. Q. Zhao X. Li Y. Zhang M. Wu Z. (2021). Evolution of the conductive filament system in HfO2-based memristors observed by direct atomic-scale imaging. *Nat. Commun.* 12:7232. 10.1038/s41467-021-27575-z 34903752 PMC8668918

[B37] Zheng J. Yu Z. Wang Y. Fu Y. Chen D. Zhou H. (2021). Acoustic core–shell resonance harvester for application of artificial cochlea based on the piezo-triboelectric effect. *ACS Nano* 15 17499–17507. 10.1021/acsnano.1c04242 34606234

[B38] Zhong S. Zhang Y. Zheng H. Yu F. Zhao R. (2022). Spike-based spatiotemporal processing enabled by oscillation neuron for energy-efficient artificial sensory systems. *Adv. Intell. Syst.* 2640–4567. 10.1002/aisy.202200076

[B39] Zhu J. Zhang X. Wang R. Wang M. Chen P. Cheng L. (2022). A heterogeneously integrated spiking neuron array for multimode-fused perception and object classification. *Adv. Mater.* 34:2200481. 10.1002/adma.202200481 35429020

