# Supplementary material for: A bioinspired configurable cochlea based on memristors
Source: Front Neurosci. 2022 Oct 3;16:982850. doi: 10.3389/fnins.2022.982850 (PMC9574047; doi:10.3389/fnins.2022.982850)
Supplement: Supplementary file 1 [file Data_Sheet_1.docx]

Supplementary Material

A bioinspired configurable cochlea based on memristors

Cheng et al.


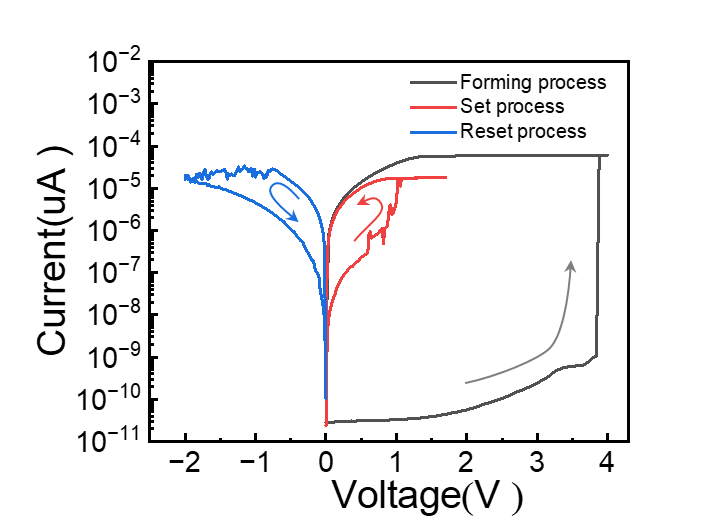


**Supplementary Figure 1.** The I-V characteristics of the 1T1R with forming/set/reset process. For forming process, a scan voltage of 0-3.7V is applied to the TE with the Vg=1.2 V; for set process, a scan voltage of 0-1.7V is applied to the TE with the V_g_=1.5V; for the reset process, a scan voltage of 0-2.2V is applied to the SE with V_g_=1.5V.

**Supplementary Figure 2.** Multilevel characteristic under DC sweep during set and reset process. When the Vg applied on gate terminal is 1V, 1.2V, 1.5V, 1.7V respectively, the corresponding scanning voltage applied on SE terminal is 0-1.65V, 0-1.8V, 0-2V, 0-2.2V respectively.


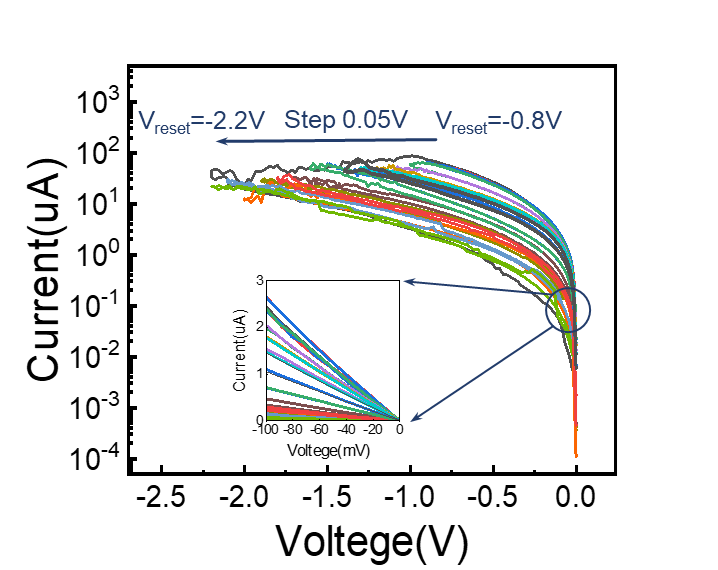


**Supplementary Figure 3.** Multilevel characteristic under DC sweep during reset process. Vreset applied on SE terminal decreases from -0.8 V to -2.2 V with a 0.05 V step to obtain multilevel memirstor state. The inset shows the good linearity of the memristor under 0-0.1 V sweeping on TE terminal.
